# A multiplexed gRNA *piggyBac* transposon system facilitates efficient induction of CRISPRi and CRISPRa in human pluripotent stem cells

**DOI:** 10.1038/s41598-020-57500-1

**Published:** 2020-01-20

**Authors:** Dane Z. Hazelbaker, Amanda Beccard, Gabriella Angelini, Patrizia Mazzucato, Angelica Messana, Daisy Lam, Kevin Eggan, Lindy E. Barrett

**Affiliations:** 1 Stanley Center for Psychiatric Research, Broad Institute of MIT and Harvard, Cambridge, MA 02142 USA; 2 Department of Stem Cell and Regenerative Biology, Harvard University, Cambridge, MA 02138 USA

**Keywords:** Genetic engineering, Pluripotent stem cells

## Abstract

CRISPR-Cas9-mediated gene interference (CRISPRi) and activation (CRISPRa) approaches hold promise for functional gene studies and genome-wide screens in human pluripotent stem cells (hPSCs). However, in contrast to CRISPR-Cas9 nuclease approaches, the efficiency of CRISPRi/a depends on continued expression of the dead Cas9 (dCas9) effector and guide RNA (gRNA), which can vary substantially depending on transgene design and delivery. Here, we design and generate new fluorescently labeled *piggyBac* (PB) vectors to deliver uniform and sustained expression of multiplexed gRNAs. In addition, we generate hPSC lines harboring AAVS1-integrated, inducible and fluorescent dCas9-KRAB and dCas9-VPR transgenes to allow for accurate quantification and tracking of cells that express both the dCas9 effectors and gRNAs. We then employ these systems to target the *TCF4* gene in hPSCs and assess expression levels of the dCas9 effectors, individual gRNAs and targeted gene. We also assess the performance of our PB system for single gRNA delivery, confirming its utility for library format applications. Collectively, our results provide proof-of-principle application of a stable, multiplexed PB gRNA delivery system that can be widely exploited to further enable genome engineering studies in hPSCs. Paired with diverse CRISPR tools including our dual fluorescence CRISPRi/a cell lines, this system can facilitate functional dissection of individual genes and pathways as well as larger-scale screens for studies of development and disease.

## Introduction

CRISPR-Cas9 systems have revolutionized genome editing in myriad cell types and organisms and ushered the development of variant technologies that utilize dCas9 fused to epigenetic modifiers which can be localized to a gene of interest upon expression of a gRNA^1–3^. Two such approaches are CRISPRi, which fuses dCas9 to transcriptional repressors, such as the KRAB domain^3^, and CRISPRa which fuses dCas9 to transcriptional activators, such as the chimeric VPR domain^2^. These tools can be deployed for both single and multiplexed gene manipulation and allow modulation of gene expression in the absence of cellular toxicity caused by Cas9-mediated DNA double-strand breaks^4^. CRISPRi/a set-ups have been used successfully in studies of cellular programming^5^, cellular reprogramming^6,7^, *in vivo* gene manipulation^8^, enhancer screens^9^, chemical screens^10^, and whole-genome genetic interaction mapping studies^11^. When targeting populations of cells, gene repression through CRISPRi is reported to be more homogeneous and efficient compared to Cas9 nuclease^12^. Indeed, while Cas9-nuclease strategies have been employed in genome-wide screens, they are limited by heterogeneity in the targeted cell populations, which may include a significant number of wild-type cells alongside cells with mixtures of indels that produce partial loss or gain of function phenotypes, or truncated gene products which can complicate interpretations^12^. Furthermore, CRISPRi/a offers the potential for conditional gene perturbation, allowing for the functional study of essential genes^3^ and reversibility of phenotypes. However, unlike genetic knockout by CRISPR-Cas9 that requires a single indel formation event to permanently disrupt gene function, successful CRISPRi/a requires persistent and uniform expression of dCas9 effectors and gRNA across cell populations, an important consideration both in single gene studies and whole-genome screens.

There is limited data on the stability of dCas9 effectors^12^ and studies report variability in the induction and expression of different promoters in different loci due to *de novo* DNA methylation^13^. Further, gRNA delivery and expression require optimization in order to fully capitalize on the multiplexing potential of CRISPRi/a. With regard to gRNA delivery, previous studies have utilized transfection and selection of plasmid DNA^12,14,15^ transient transfection of *in vitro* transcribed gRNA^16,17^, lentiviral integration^17^ or *piggyBac* transposon-based integration^18^. In particular, *piggyBac* (PB) delivery methods have the advantages of being easy to clone and deliver into hPSCs and carry substantially larger payload compared to lentiviral vectors^19,20^. As a result, PB vectors are particularly applicable for studies of parallel pathways or polygenic disease, enabling the perturbation of many genes with a single delivery vehicle at minimal cost.

Here, we developed a new *piggyBac* vector system to enable rapid cloning and stable delivery of multiple gRNAs for CRISPRi/a applications. We coupled this system with a set of hPSC lines harboring genomically integrated and inducible dCas9-KRAB and dCas9-VPR, including a dual-fluorescent readout to readily quantify cells that express both gRNAs and dCas9 variants in a population. We then quantified expression levels of the effector components as well as a targeted gene, *TCF4*, at both the transcript and protein levels. We also assessed the performance of our PB system for single gRNA delivery targeting *TCF4* and *POGZ*. Our results confirm the utility of the dual-fluorescent readout and multiplexed or single PB gRNA delivery system for CRISPRi/a that can be broadly employed in hPSCs for gene perturbation studies.

## Results

### Generation of AAVS1 integrated and doxycycline-inducible dCas9-KRAB and dCas9-VPR hPSC lines

**Figure 1:**
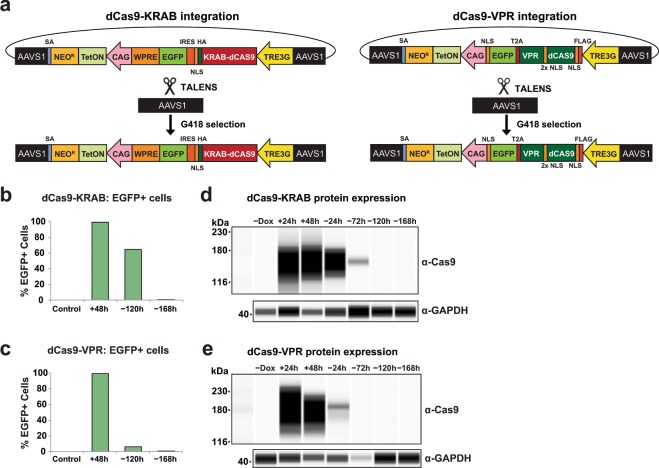
Generation and validation of AAVS1-integrated inducible dCas9-KRAB and dCas9-VPR hPSC lines. (**a**) Schematic overview of AAVS1 targeting strategy in H1 hPSCs with TRE3G-driven dCas9-KRAB (left) or dCas9-VPR (right) cassettes and TALENs that target AAVS1 and confer G418 resistance upon on-target integration. (**b**) Quantification of flow cytometry analysis of EGFP fluorescence in dCas9-KRAB cells after 48 hours of doxycycline treatment (+48 h) followed by removal of doxycycline for 120 (−120 h) and 168 hours (−168 h) in comparison to no GFP control H1 cells (control). (**c**) Quantification of flow cytometry analysis of EGFP fluorescence in dCas9-VPR cells after 48 hours of doxycycline treatment (+48 h) followed by removal of doxycycline for 120 (−120 h) and 168 hours (−168 h) in comparison to no GFP control H1 cells (control). (**d**) dCas9-KRAB protein expression in absence of doxycycline (−Dox), after 24 and 48 hours doxycycline treatment (+24 h, +48 h), and after washout of doxycycline for 24, 72, 120, and 168 hours (−24 h, −72 h, −120 h, −168 h). (**e**) dCas9-VPR protein expression before doxycycline treatment (-Dox), after 24 and 48 hours doxycycline treatment (+24 h, +48 h) and after washout of doxycycline for 24, 72, 120, and 168 hours (−24 h, −72 h, −120 h, −168 h). Images in **d,e** derived from Wes analysis (ProteinSimple).

To create stable CRISPRi and CRISPRa hPSC lines, we cloned and introduced all-in-one cassettes containing *S. pyogenes* dCas9 fused to the KRAB repressor domain^21^ or VPR activation domain^2^ into the AAVS1 safe-harbor locus of the XY embryonic stem cell line H1^22^ via a TALEN-mediated gene-trap approach that confers neomycin (G418) resistance to cells upon on-target integration^12,16^ (Fig. 1a). In both constructs, dCas9-KRAB and dCas9-VPR expression is driven by the TRE3G doxycycline inducible promoter (Takara Bio) and fused to Enhanced Green Fluorescent Protein (EGFP) transcriptional reporters by an IRES sequence (dCas9-KRAB) or a T2A self-cleaving peptide sequence (dCas9-VPR). Following selection with G418, dCas9-KRAB and dCas9-VPR clones were assessed for EGFP expression and genotyped by junction PCR (Supplementary Fig. S1). From these data, dCas9-KRAB and dCas9-VPR clones were expanded and confirmed to have normal karyotypes (data not shown).

To validate inducible and reversible dCas9 expression in our CRISPRi/a hPSC lines, we first quantified EGFP fluorescence by flow cytometry following 48 hours of doxycycline treatment. Doxycycline led to strong induction of EGFP fluorescence, reaching 99% in both dCas9-VPR and dCas9-KRAB hPSC lines (+48 h; Figs. 1b,c, Supplementary Fig. S1). 168 hours after washout of doxycycline, EFGP fluorescence levels dropped to background levels in both the dCas9-KRAB and dCas9-VPR lines (−168 h; Fig. 1b,c, Supplementary Fig. S1). We also observed strong induction of dCas9-KRAB and dCas9-VPR protein expression after doxycycline induction (Fig. 1d,e, Supplementary Fig. S1) and loss of detectable dCas9 expression by 120 hours post-washout in dCas9-KRAB cells and by 72 hours in dCas9-VPR cells (Fig. 1d,e, Supplementary Fig. S1). The increased stability of dCas9-KRAB and EGFP protein in dCas9-KRAB cells in comparison to dCas9-VPR cells may be due to the presence of the WPRE (Woodchuck Hepatitis Virus Post-transcriptional Response Element) in the 3′UTR of the dCas9-KRAB construct (Fig. 1a), which has been reported to increase transcript stability^23^. Similar to previous reports, dCas9 protein was not detected in the absence of doxycycline^12^ (Fig. 1d,e, Supplementary Fig. S1). These data confirm that our AAVS1-integrated dCas9-KRAB and dCas9-VPR constructs exhibit robust induction and reversibility of dCas9 expression in hPSCs.

### Identification of the relevant transcriptional start site of *TCF4* in hPSCs

**Figure 2:**
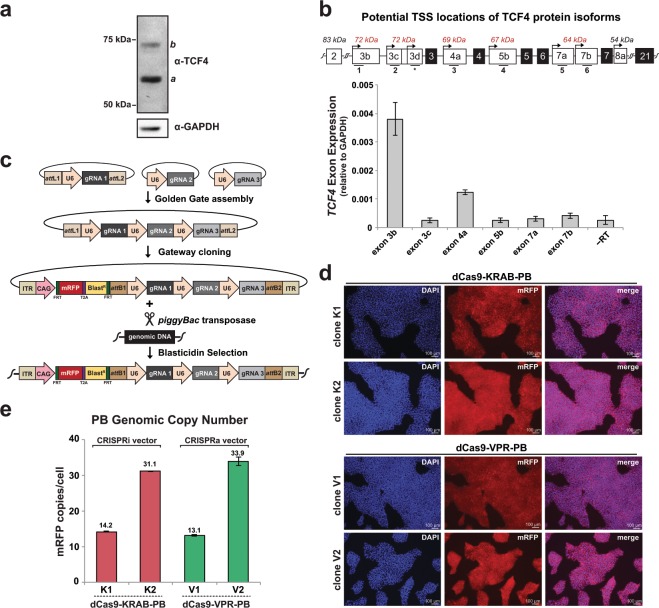
Design and delivery of multi-gRNA PB vectors for CRISPRi and CRISPRa targeting of the *TCF4* gene. (**a**) Representative western blot of TCF4 protein expression in hPSCs. Letters *a* and *b* denote the two observed bands. **(b**) *Top*, Schematic of the *TCF4* gene exons containing potential TSS locations for the putative protein isoforms shown in **(a)**. Numbers below exons correspond to the primer pairs used for RT-qPCR. Exon locations are modified from Sepp *et al*.^27^, with approximate predicted molecular weights of resulting protein isoform(s) listed above each starting exon in italics, with tested exons highlighted in red. The asterisks under exon 3d indicates there was no detectable PCR signal for this exon with two separate primer pairs (data not shown). *Bottom*, Exon-specific expression of *TCF4* transcripts from RT-qPCR of two independent H1 hPSC samples and normalized to GAPDH. -RT lane denotes the no reverse transcriptase control cDNA reaction. Data is shown as mean +/− s.e.m. of two independent biological samples. (**c**) Overview of multi-gRNA PB vector cloning, delivery, and selection. (**d**) Representative images of mRFP fluorescence in dCas9-KRAB-PB clones K1 and K2 (*top panels*) and dCas9-VPR-PB clones V1 and V2 (*bottom panels*). Cells are counterstained with DAPI (blue). Scale bar = 100 μm. (**e**) PB vector copy number in dCas9-KRAB and dCas9-VPR clones as determined by ddPCR quantification of mRFP gene. Data is shown as the mean of three experiments with error bars as +/− s.e.m.

To assess the potency of our dCas9-KRAB and dCas9-VPR systems for gene repression and activation, we chose to target the *TCF4* gene in hPSCs as an example. *TCF4* plays important roles in development and *TCF4* gene dysfunction has been implicated in multiple neurodevelopmental diseases including Pitt-Hopkins syndrome and schizophrenia by GWAS^24–26^. Importantly, *TCF4* has multiple alternatively-spliced transcripts^27^ making it critical to identify the most relevant TCF4 isoform and its corresponding transcriptional start site (TSS) to target with CRISPRi/a. Generally speaking, functional gRNA design for CRISPRi/a applications has the added challenge that TSSs may not be well annotated for a given cell type. We therefore first carried out western blot analysis with a recombinant antibody (ab217668, Abcam) that targets a fragment present in all known TCF4 isoforms (amino acids 350–500) and observed two protein bands at approximately 62 kDa (band *a*) and 72 kDa (band *b*) (Fig. 2a, Supplementary Fig. S2). To determine the TSSs of potential transcripts that upon translation could generate peptides within the size range of the observed bands in Fig. 2a, we utilized exon-specific RT-qPCR in H1 hPSCs with primers targeting candidate TSS-harboring exons. (Fig. 2b). As shown in Fig. 2b, RT-qPCR analysis revealed exon 3b to be the most dominantly expressed exon, which is the leading exon of the *TCF4* 3b transcript. Translation of the *TCF4* 3b transcript is reported to generate the canonical TCF4-B isoform^27^ of approximately 72 kDa corresponding to band *b* in Fig. 2a. Importantly, the FANTOM5 promoterome (fantom.gsc.riken.jp/zenbu), which is commonly used to identify the TSS for gRNA design^28,29^, annotated exon 3d as harboring the dominant TSS based on pooled CTSS tracks (Supplementary Fig. S2). However, we did not observe detectable expression of exon 3d via RT-qPCR in H1 hPSCs using two different primer pairs (data not shown, primers listed in Supplementary Table S1) thus underscoring the importance of validating the relevant TSS for a given cell type in single gene studies. Thus, taking into account our protein and transcript analysis above, we opted to target exon 3b and designed three *TCF4* CRISPRi gRNAs (i1, i2, i3) and three *TCF4* CRISPRa gRNAs (a1, a2, and a3) that fall within the optimal window of 300 bp from the TSS^3,29^ using the CRISPR-ERA guide selection tool^30^ (Supplementary Fig. S2).

### Design and delivery of multi-gRNA *piggyBac* vectors in hPSCs

A noted strength of CRISPRi and CRISPRa is the ability to deliver multiple gRNAs for enhanced targeting of one or several genes in the absence of DNA damage^31^. To facilitate stable delivery of multiple gRNAs in hPSCs, we designed a new vector that incorporates the efficiency and ease of the *piggyBac* (PB) transposase system^32^ with a multiplex gRNA cloning system^33^. To do so, we cloned either the three CRISPRi gRNAs (i1, i2, i3) or CRISPRa gRNAs (a1, a2, a3) targeting *TCF4* into individual vectors and sequentially assembled the final PB vector including mRFP and blasticidin resistance via Golden Gate and Gateway cloning (Fig. 2c). Of note, while we opted to introduce three gRNAs per PB vector, the parental vectors allow for the cloning of up seven gRNAs in tandem array^33^ that can readily be introduced into our PB vectors. Additionally, *FRT* sites flank the mRFP and blasticidin cassettes (Fig. 2c) to facilitate removal of these selectable features with FLP recombinase and allow for future PB re-targeting events. Using our optimized workflow, multiple gRNAs can be cloned into PB vectors and confirmed via BamHI restriction digest within one week (Supplementary Fig. S2 and Methods).

We next co-transfected the multi-gRNA PB vectors along with a plasmid encoding the *piggyBac* transposase into dCas9-KRAB and dCas9-VPR hPSC lines. Following selection with blasticidin, individual dCas9-KRAB and dCas9-VPR clones were isolated and screened for high levels of uniform mRFP fluorescence (Fig. 2d). We then selected and expanded two independent dCas9-KRAB clones (dCas9-KRAB-PB clones K1 and K2) and two independent dCas9-VPR clones (dCas9-VPR-PB clones V1 and V2) and assessed integrated PB copy number via droplet digital PCR (ddPCR) of the mRFP cassette in both the CRISPRi and CRISPRa PB vectors. Our ddPCR analysis revealed approximately 14 and 31 PB copies in dCas9-KRAB-PB clones K1 and K2, respectively, and approximately 13 and 34 PB copies dCas9-VPR-PB clones V1 and V2, respectively (Fig. 2e). We also confirmed that both dCas9-KRAB-PB and dCas9-VPR-PB cells harboring moderate levels of integrated PBs (i.e., 13–34 copies) maintained pluripotency and tri-lineage potential (Supplementary Fig. S2). Thus, our new PB vectors facilitate both rapid cloning and efficient delivery of multiple gRNAs into hPSCs.

### Quantification of CRISPRi/a component expression in hPSCs

**Figure 3:**
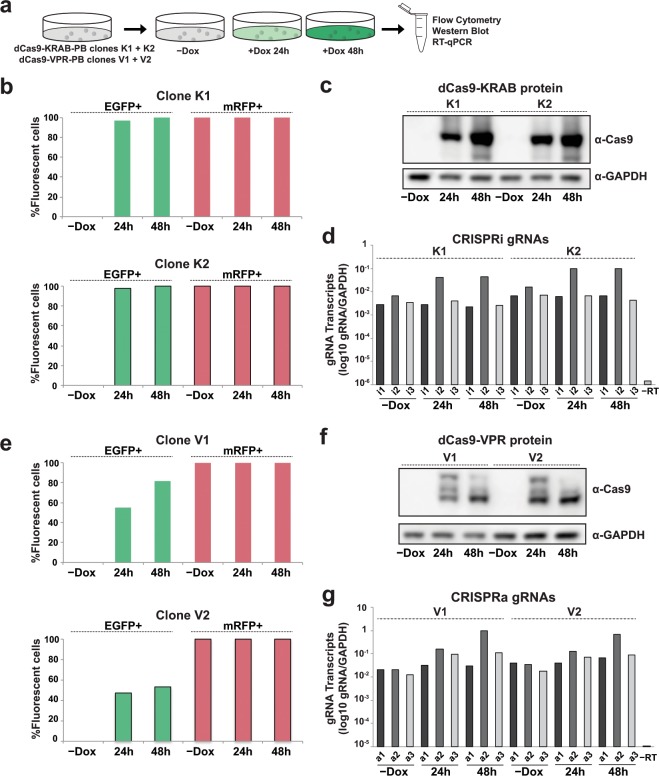
Assessment of CRISPRi and CRISPRa component expression. (**a**) Experimental overview for activation and repression of *TCF4* in dCas9-KRAB-PB and dCas9-VPR-PB clones **(b**) Quantification of flow cytometry analysis of EGFP and mRFP fluorescence in dCas9-KRAB-PB clones K1 and K2 in absence of doxycycline (−Dox) and in presence of doxycycline for 24 and 48 hours. (**c**) Western blot analysis of dCas9-KRAB protein in dCas9-KRAB-PB clones K1 and K2 at indicated time-points. (**d**) RT-qPCR analysis of CRISPRi gRNAs i1, i2, and i3 in dCas9-KRAB-PB clones K1 and K2. (**e**) Quantification of flow cytometry analysis of EGFP and mRFP fluorescence in dCas9-VPR-PB clones V1 and V2 at indicated time-points. (**f**) Western blot analysis of dCas9-VPR protein level in dCas9-VPR-PB clones at indicated time-points. (**g**) RT-qPCR analysis of CRISPRa gRNAs a1, a2, and a3 expression in dCas9-VPR-PB clones V1 and V2.

To quantify expression levels of both the dCas9 effector and individual gRNAs, we treated dCas9-KRAB-PB and dCas9-VPR-PB clones with doxycycline for 0, 24, and 48 hours and collected replicate and matched samples for side-by-side clonal analysis via flow cytometry, western blot, and RT-qPCR (Fig. 3a). For CRISPRi, flow cytometric quantification of EGFP fluorescence of the two independent dCas9-KRAB-PB clones showed high levels of EGFP fluorescence (99.9% for both K1 and K2 clones) and mRFP expression fluorescence (100% for both K1 and K2 clones) after 48 hours of doxycycline induction (Fig. 3b, Supplementary Fig. S3) indicating robust and uniform expression of dCas9-KRAB and gRNA. In direct congruence with the EGFP and mRFP fluorescence data, we observe strong induction of dCas9-KRAB protein (Fig. 3c, Supplementary Fig. S3) and high levels of all three CRISPRi gRNAs by gRNA-specific RT-qPCR (Fig. 3d) in both K1 and K2 clones. Indeed, we observed gRNA expression levels between 1% and nearly 100% of the levels of *GAPDH* transcripts. These results indicate that PB vectors provide a consistent and reproducible means to express multiple gRNAs across cells in a population using a single delivery vehicle.

In the case of CRISPRa, flow cytometric quantification of EGFP fluorescence of the two independent dCas9-VPR-PB clones revealed intermediate levels of EGFP fluorescence (82% for clone V1 and 53% for clones V2; Fig. 3e, Supplementary Fig. S3). The decreased levels of EGFP expression in dCas9-VPR-PB clones contrasts with the high levels of EGFP expression in the parental dCas9-VPR clone (Fig. 1c), perhaps indicating a CRISPRa gRNA- or PB-specific effect as prolonged doxycycline treatment in dCas9-VPR cells without integrated PB vectors did not result in reduced EGFP levels (data not shown). However, mRFP expression remained high in dCas9-VPR-PB cells, as quantified by flow cytometry (100% for both clone V1 and V2). Direct assessment of dCas9-VPR protein levels in clones V1 and V2 revealed strong induction upon doxycycline treatment (Fig. 3f, Supplementary Fig. S3), albeit to a lesser extent compared with dCas9-KRAB-PB clones. Again, RT-qPCR analysis showed robust and uniform expression of all three CRISPRa gRNAs (Fig. 3g) further confirming the multiplexing capacity of the PB system. Interestingly, the expression levels of CRISPRi and CRISPRa gRNAs i2 and a2 increased 4–6 fold upon expression of dCas9 by doxycycline treatment (Fig. 3d,g). It is possible that the presence of dCas9 selectively increases gRNA stability by binding particular gRNAs with high affinity and protecting them from degradation, perhaps by masking the 5′ end of the gRNA, as suggested by previous studies^34^. These results demonstrate that both dCas9-effectors and multiplex gRNAs are efficiently expressed in our CRISPRi and CRISPRa hPSC lines.

### Quantification of TCF4 repression and activation at the transcript and protein level

**Figure 4:**
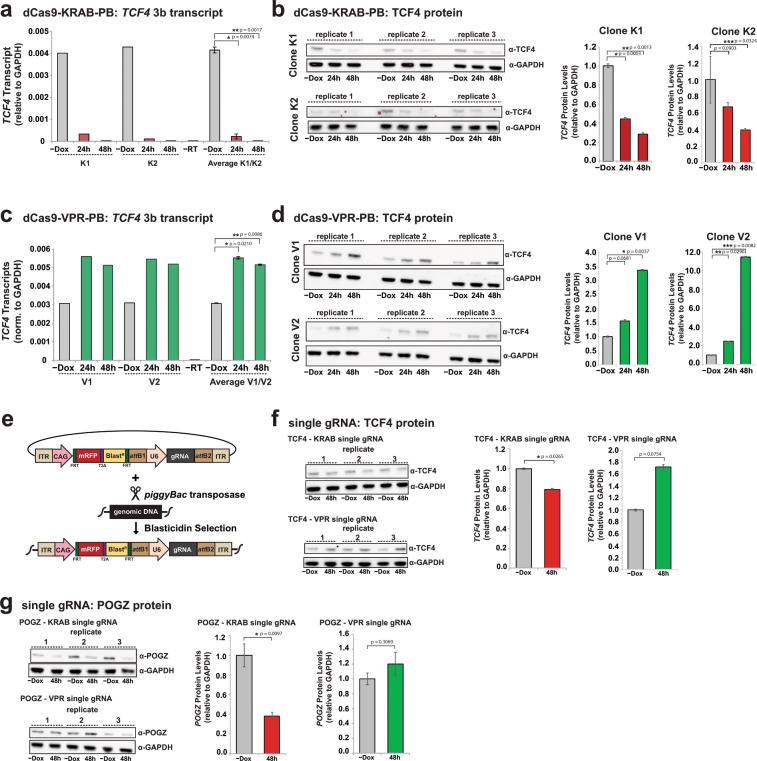
Gene repression and activation at the transcript and protein levels. (**a**) RT-qPCR analysis of *TCF4* 3b transcript levels at indicated time points in dCas9-KRAB-PB clones K1 and K2 separately and averaged. Data is shown as mean +/− s.e.m. *p = 0.0074, **p = 0.0017. (**b**) Western blot analysis of TCF4 protein in dCas9-KRAB-PB clones K1 and K2 in biological triplicate at indicated time-points with quantification shown on the right. *p = 0.0003, **p = 0.0013 and ***p = 0.0324. (**c**) RT-qPCR analysis of *TCF4* 3b transcript levels at indicated time points in dCas9-VPR-PB clones V1 and V2 separately and averaged. Data is shown as mean +/− s.e.m. *p = 0.0210, **p = 0.0086. (**d**) Western blot analysis of TCF4 protein in dCas9-VPR-PB clones V1 and V2 in biological triplicate at indicated time-points with quantification shown on the right. *p = 0.0037, **p = 0.02901, ***p = 0.0082. (**e**) Overview of single-gRNA PB vector cloning, delivery, and selection. (**f**) Western blot analysis of TCF4 protein with single gRNAs in dCas9-KRAB and dCas9-VPR cells in biological triplicate with quantification shown on the right. *p = 0.0265. (**g**) Western blot analysis of POGZ protein with single gRNAs in dCas9-KRAB and dCas9-VPR cells in biological triplicate with quantification shown on the right. *p = 0.0097. In all Western blot panels, expression levels are normalized to GAPDH.

Having established robust expression of our effector components, we next sought to quantify levels of repression and activation of a target gene in hPSCs. To quantify the efficiency of *TCF4* repression by CRISPRi, we first analyzed *TCF4* 3b transcript levels by RT-qPCR in dCas9-KRAB-PB clonal pairs. As shown in Fig. 4a, doxycycline induction resulted in rapid and significant repression of TCF4 transcripts in both clones K1 and K2 (average decrease of 18-fold at 24 hours and 200-fold at 48 hours). Comparison of TCF4 transcript levels in clone K1 and K2 revealed that clone K2 displayed more rapid repression, suggesting that CRISPRi potency may titrate with PB copy number (Fig. 2e). As expected, TCF4 protein was significantly reduced in both dCas9-KRAB K1 and K2 clones by 48 hours (Fig. 4b, Supplementary Fig. S4). By comparison, targeting of *TCF4* for activation in dCas9-VPR-PB clones (V1 and V2) resulted in a 1.8- and 1.6-fold average increase in transcript levels after 24 hours and 48 hours doxycycline treatment, respectively (Fig. 4c). In dCas9-VPR-PB cells TCF4 protein levels were also significantly increased after 48 hours of doxycycline induction (Fig. 4d, Supplementary Fig. S4).

### Assessing compatibility for single gRNA delivery

To assess the performance of our PB system for delivering single gRNAs, such as those that might be used in library format, we cloned individual PB vectors containing one CRISPRi gRNA targeting *TCF4*, one CRISPRa gRNA targeting *TCF4*, one CRISPRi gRNA targeting *POGZ*, or one CRISPRa gRNA targeting *POGZ* (Fig. 4e). We then co-transfected the single-gRNA PB vectors along with a plasmid encoding the *piggyBac* transposase into the relevant dCas9-KRAB and dCas9-VPR hPSC lines. Following selection with blasticidin and expansion, cells were treated with doxycycline for 0 or 48 hours for western blot analysis (Fig. 4f,g, Supplementary Fig. S4). TCF4 and POGZ protein levels were each significantly reduced using a single gRNA in dCas9-KRAB cells (Fig. 4f,g, Supplementary Fig. S4). In the case of dCas9-VPR cells, TCF4 and POGZ protein levels both trended toward an increase, although the results did not reach statistical significance. The variability in gene modulation within each system is likely due to the relative efficiencies of individual gRNAs. As the single TCF4 gRNAs (Fig. 4f) were also included in the multiplexed system (Fig. 4b,d) these data also indicate that the inclusion of additional gRNAs can lead to additional gene modulation.

## Discussion

CRISPRi/a systems hold great potential for exploring gene function and dissecting human disease mechanisms in hPSCs and hPSC-derived cell types, such as cardiomyocytes^12^ and neurons^17^. Benefits of CRISPRi/a over knockout strategies utilizing Cas9 nuclease include the ability to conditionally perturb essential and multiple genes in the absence of DNA damage and genetic instability^35,36^. However, in contrast to gene perturbation by gene knockout with CRISPR-Cas9, gene modulation by CRISPRi and CRISPRa approaches are dependent on sustained expression of the dCas9 effector and gRNA.

Here, we developed a set of tools to facilitate multiplexed CRISPR-mediated gene modulation in hPSCs. We find that our integrated dCas9-KRAB and dCas9-VPR constructs allow for reproducible and reversible induction of dCas9 alongside EGFP in the vast majority of cells, consistent with previous reports^12^. To facilitate stable and multiplex gRNA expression, we designed and validated a drug-selectable *piggyBac* vector with constitutive mRFP fluorescence to visualize and track gRNA-expressing cells. Thus, dual monitoring of both EGFP and mRFP fluorescence allows for quantification of the percentage of CRISPRi/a competent cells in a population.

With regard to gRNA expression, we find that CAG-promoter driven PB vectors support sustained gRNA and reporter expression in hPSCs. Importantly, we confirmed high expression levels of six independently cloned gRNAs by direct assessment via RT-qPCR in the multiplexed system, demonstrating that PB vectors provide a dependable, rapid and inexpensive delivery vehicle for transgene expression. Specifically, we anticipate these vectors will be useful for rapid and multiplexed expression of gRNAs in hPSCs for perturbation analysis at the single gene and whole genome levels, both in CRISPRi/a contexts, as presented here, and in CRISPR knockout schemes with Cas9 nuclease or Cas9-fused base editors^37^. Indeed, our dCas9-VPR construct and PB vectors have been successfully employed in another hPSC line, 18a, to target the *XIST* gene (Fukuda *et al*., unpublished results), supporting the reproducibility of our system with additional cell lines and gene targets.

Collectively, our newly designed gRNA PB vectors deliver robust, sustained gRNA expression in hPSCs. Further, the coupling of these tools with our dual-fluorescence dCas9-KRAB and dCas9-VPR systems facilitates accurate quantification and tracking of CRISPRi/a components across cells in a population. We anticipate these tools will facilitate both single and multiplexed gene perturbation studies and screens in hPSCs and other cell types for functional interrogation of development and disease.

## Methods

### Plasmid construction

To generate the dCas9-KRAB-IRES-EGFP AAVS1 targeting plasmid pT077, parental plasmid pHR-TRE3G-KRAB-dCas9-IRES-GFP (a gift from Jesse Engreitz, Broad Institute) was cloned by Gibson assembly into the backbone fragment of plasmid pGEP116 that contains AAVS1 homology arms and the doxycycline-responsive activator rTTA driven by a constitutive CAG promoter^38^. To generate the dCas9-VPR-T2A-EGFP AAVS1 targeting plasmid (pT076), the dCas9-VPR cassette from plasmid SP-dCas9-VPR^2^ (Addgene 63798) was fused by Gibson assembly with a PCR fragment containing a T2A-EGFP-NLS cassette (from plasmid PT059) and cloned into plasmid pGEP116. Oligonucleotides (IDT) corresponding to gRNA target sequences (Supplemental Table S1) were cloned via BpiI into pX330S-2 and pX330S-3 and a third vector pGEP179_pX330K (this study) according to kit instructions^33^ (Addgene Kit#1000000055). The pGEP179_pX330K plasmid is a modified entry vector generated by cloning the BsaI-pU6-sgRNA-BsaI fragment from pX330A-1 × 3^33^ into a slightly modified MCS of the *att*L-containing entry vector, pENTR1A (Invitrogen). These gRNA-containing pX330S and pGEP179_pX330K plasmids were then assembly by Golden Gate cloning to form a single entry vector. This entry vector was then cloned by Gateway cloning into the *piggyBac* donor destination plasmid pGEP163 that contains *piggyBac* ITRs for transposase-mediated insertion, a CAG promoter driving an mRFP-T2A-BLAST^R^ cassette, and *att*R sites Gateway cloning to create CRISPRi multi-gRNA plasmid pPN441 and CRISPRa multi-gRNA plasmid pPN440. Donor plasmid pGEP163 was constructed by fusing a fragment of plasmid PB-CA^39^ (Addgene 20960) containing the *piggyBac* ITRs and a CAG promoter with a synthetic gene block containing FRT-mRFP-T2A-BLAST^R^-SV40 pA-FRT (IDT). The versions of pPN441 and pPN440 plasmids used in this study were initially created by an earlier cloning strategy that was replaced by the strategy described above to generate the same pPN441 and pPN440 plasmids more rapidly. BamHI digests of pPN441 and pPN440 were performed according to manufacturer’s instructions (New England Biolabs). Single gRNAs were cloned into pGEP179_pX330K and this entry vector was then cloned by Gateway cloning into the *piggyBac* donor destination plasmid pGEP163, as described above, for generation of the final pPN458 (TCF4 VPR), pPN459 (TCF4 KRAB), pPN460 (POGZ VPR) and pPN461 (POGZ KRAB) constructs.

### Cell culture and gene targeting

The human embryonic stem cell line H1^40^ (WA01) was obtained commercially from WiCell Research Institute (Madison, WI). The use of H1 was approved by Harvard University’s ESCRO committee and methods were carried out in accordance with approved guidelines. Stem cells were grown in either mTeSR1 medium (Stem Cell Technologies 05850) or StemFlex medium (ThermoFisher A3349401) on Geltrex (Life Technologies A1413301) coated plates under conditions previously described^41^. Throughout culturing, cells were tested to confirm the absence of mycoplasma contamination (Lonza MycoAlert LT07-418). To integrate the dCas9-KRAB and dCas9-VPR constructs into the AAVS1 locus, 2.5 × 10^6^ cells were co-transfected with 10ug of pT077 (KRAB) or pT076 (VPR), 1.5 µg AAVS1 TALEN L (Addgene 59025) and 1.5 µg AAVS1 TALEN R (Addgene 59026) via the Neon Electroporation System (ThermoFisher) at 1050 mV, 30 ms, 2 pulses. For the first round of clonal selection, the transfected cells were plated at low-density (8,000 cells in a 10 cm dish) under G418 selection (50 ug/ml, Gibco 10131035) to allow for single-cell colony formation (~10 days). Importantly, cells with the dCas9-KRAB and dCas9-VPR cassettes are kept under selection with G418 for the duration of culture and experiments to protect against shutdown of the AAVS1 integrated transgenes. In this strategy, colonies are picked and deposited into a 96-well plate and when sufficiently dense, the 96-well plate is triplicated to create 3 plates of identical clones. Plate 1 is frozen for storage, plate 2 is treated with doxycycline (Sigma, D9891-25g) at a final concentration of 2 µg/ml 24 hours after duplication for visualization of EGFP + colonies (with high levels of EGFP expression serving as a proxy for high dCas9 expression), and plate 3 is maintained for expansion and banking of EGFP + colonies (n = 6) while the analysis of plate 2 is performed. For integration of the multiplex PB vectors, 2.5 × 10^6^ dCas9-KRAB and dCas9-VPR cells were transfected with 5 µg of pPN441 (CRISPRi multi-gRNA plasmid) and 5 µg of pPN440 (CRISPRa multi-gRNA plasmid), respectively, with 1 µg of transposase plasmid (System Biosciences #PB210PA-1) under conditions described above. 24 hours after transfection, cells are treated with blasticidin at a final concentration of 2 µg/ml for 12–15 days to select for positive *piggyBac* integrants and allow clearing of free plasmid. Genomic DNA for PCR-based genotyping and *piggyBac* copy number analysis by ddPCR was isolated via the DNeasy Blood and Tissue Kit (Qiagen 69504). For doxycycline induction of dCas9-KRAB and dCas9-VPR, cells are treated with 2 µg/ml doxycycline and pelleted at indicated time points.

### Western blot analysis

To isolate protein for western blot analysis, hPSCs were lysed using Pierce IP lysis buffer (Life Technologies 87787) with protease inhibitors (Sigma Aldrich 11836153001). 20 μg total protein, as determined by Pierce BCA Protein Assay kit (Thermo Scientific 23227), was loaded onto Bolt 4–12% NuPAGE Bis-Tris Plus gels (Invitrogen). Gels were transferred overnight at 4 °C to nitrocellulose membranes in 1X NuPAGE transfer buffer (Invitrogen) with 10% methanol. The following antibodies were used for western blot analysis: Cas9 (Diagenode C15310258, 1:1000) TCF4 (Abcam ab217668, 1:500), POGZ (Abcam ab167408, 1:1000), GAPDH (EMD MAB374; 1:2000), α-rabbit HRP-linked F(ab’)2 (GE Life Sciences NA9340; 1:5000) and α-mouse HRP-linked F(ab’)2 (GE Life Sciences NA9310; 1:5000). Blots were visualized by chemiluminescence with the SuperSignal West Femto kit (Pierce) and imaged and quantified with a ChemiDoc MP Imaging System (BioRad). For quantification of Cas9 protein in dCas9-KRAB and dCas9-VPR parental clones, 1.2 μg total protein was analysed with Cas9 (Diagenode C15310258, 1:400) and GAPDH (EMD MAB374; 1:50) antibodies using the Wes capillary immunoassay system (ProteinSimple).

### Flow cytometry

Flow cytometry was performed at the Broad Institute Flow Facility on a CytoFLEX flow cytometer (Beckman Coulter). Cells were treated with 10 mM ROCK inhibitor (Y-27632) for 4 to 6 hours prior to analysis. For each experiment, 100,000 events were recorded and analyzed with FCS Express 6 software (De Novo Software).

### Genomic DNA isolation and genotyping PCR and ddPCR

Genomic DNA (gDNA) was extracted from hPSCs with the DNeasy Blood and Tissue kit according to manufacturer’s instructions (Qiagen). For genotyping of WT AAVS1 in dCas9-KRAB and dCas9-VPR clones, PCR of gDNA was performed with primer pair GE222 and GE668. For genotyping of gene targeted AAVS1 in dCas9-KRAB cells, PCR was performed with primer pair GE222 and GE586 for 5′ junctions and primer pair GE819 and GE668 for 3′ junctions. For genotyping gene targeted AAVS1 in dCas9-VPR cells, PCR was performed with primer pair GE222 and GE332 for 5′ junctions and primer pair GE233 and GE668 for 3′ junctions. For ddPCR of gDNA to quantify *piggyBac* copy number in dCas9-KRAB-PB and dCas9-VPR-PB clones, 20 μl reactions were prepared with ddPCR Supermix for Probes (no dUTP) (Bio-Rad, #1863024) with probes specific to mRFP and control gene *RPP30* according to manufacturer’s instructions (Bio-Rad). Droplets were generated using a QX100 Droplet Generator and PCR was performed on a C1000 Touch thermal-cycler (Bio-Rad) followed by sample streaming onto a QX100 Droplet Reader (Bio-Rad). Quantification was performed with QuantaSoft software. Primer sequences are listed in Table S1.

### RT-qPCR

Total RNA from hPSCs was extracted using an RNeasy Mini Kit (Qiagen). Reverse Transcription cDNA synthesis reactions were performed on 0.2–2 μg total RNA with iScript cDNA synthesis kit (BioRad) according to manufacturer’s instructions. Quantitative PCR reactions were performed the iTaq Universal SYBR Green Supermix (BioRad) and quantified by the ΔΔcT method on a CFX384 Real-Time System (Bio-Rad). All primers that yielded detectable PCR product had amplification efficiencies between 95–110%. Primer sequences are listed in Table S1.

### Embryoid body differentiation and immunostaining

Embryoid bodies (EBs) were generated as previously described^41^. For immunostaining, hPSC colonies and EBs were fixed with 4% paraformaldehyde in PBS for 15 mins at room temperature (RT), blocked and permeabilized with 0.1% TritonX-100 and 4% serum in PBS for 1 hr at RT and incubated with the appropriate primary antibody at RT. Following primary antibody incubation, cells were washed with PBS and incubated with the appropriate secondary antibody (Alexa Fluor 488 or 594, 1:500, Invitrogen) for 1 hr. Cells were then washed with PBS and incubated with DAPI before imaging at 20X magnification. The following primary antibodies were used: OCT4 (R&D Systems AF1759; 1:250), SSEA-4 (SCBT SC21704; 1:250), TRA-1-60 (SCBT SC21705; 1:200), AFP (Sigma A8452; 1:250), SMA (Sigma A2547; 1:2000), β-III Tubulin (R&D Systems MAB1195; 1:3000).

## Supplementary information

Supplementary Information.

## Data Availability

All plasmids generated in this study including all-in-one dCas9-KRAB and dCas9-VPR targeting plasmids and multiplexed PB gRNA delivery systems will be deposited in Addgene.org. Cell lines will be made available upon request with appropriate institution approvals and following WiCell requirements for cell line distribution.
