# Supplementary material for: A multiplexed gRNA piggyBac transposon system facilitates efficient induction of CRISPRi and CRISPRa in human pluripotent stem cells
Source: Sci Rep. 2020 Jan 20;10:635. doi: 10.1038/s41598-020-57500-1 (PMC6971260; doi:10.1038/s41598-020-57500-1)
Supplement: Supplementary file 1 — Supplementary Information. [file 41598_2020_57500_MOESM1_ESM.pdf]

Supplementary Information

A multiplexed gRNA *piggyBac* transposon system facilitates efficient induction of CRISPRi and CRISPRa in human pluripotent stem cells

Dane Z. Hazelbaker, Amanda Beccard, Gabriella Angelini, Patrizia Mazzucato, Angelica Messana, Daisy Lam, Kevin Eggan and Lindy E. Barrett

Contents:

Figure S1
Figure S2
Figure S3
Figure S4
Supplementary Figure Legends
Supplementary Table S1

FIGURE S1

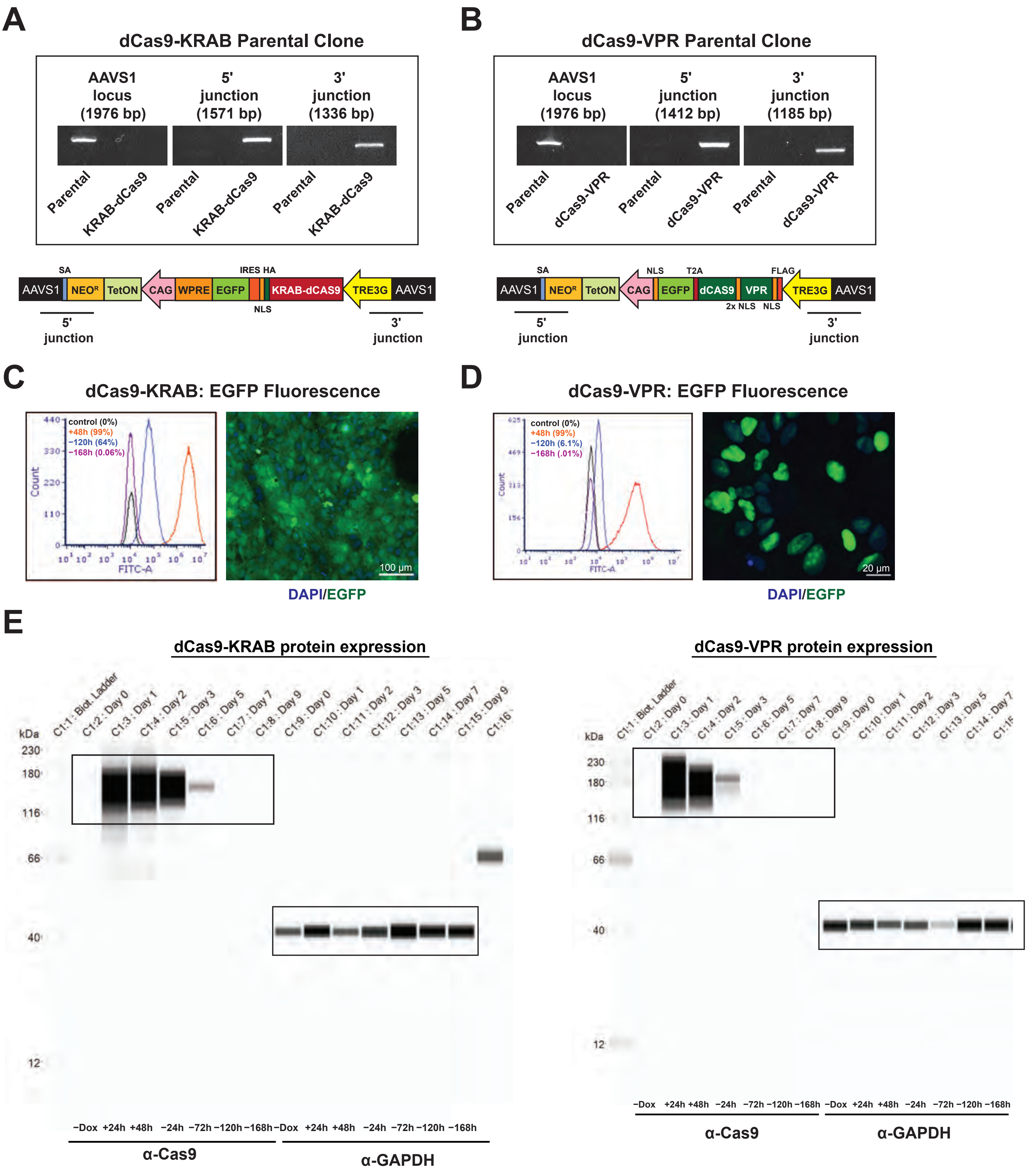
A
dCas9-KRAB Parental Clone
AAVS1 locus (1976 bp)
5' junction (1571 bp)
3' junction (1336 bp)
Parental
KRAB-dCas9
SA
IRES HA
AAVS1
NEO^R
TetON
CAG
WPRE
EGFP
KRAB-dCAS9
TRE3G
NLS
5' junction
3' junction
B
dCas9-VPR Parental Clone
AAVS1 locus (1976 bp)
5' junction (1412 bp)
3' junction (1185 bp)
Parental
dCas9-VPR
SA
NLS
T2A
FLAG
dCAS9
VPR
2x NLS
C
dCas9-KRAB: EGFP Fluorescence
control (0%)
+48h (99%)
−120h (64%)
−168h (0.06%)
Count
FITC-A
100 µm
DAPI/EGFP
D
dCas9-VPR: EGFP Fluorescence
control (0%)
+48h (99%)
−120h (6.1%)
−168h (.01%)
20 µm
E
dCas9-KRAB protein expression
dCas9-VPR protein expression
kDa
230
180
116
66
40
12
−Dox +24h +48h −24h −72h −120h −168h
α-Cas9
α-GAPDH

FIGURE S2

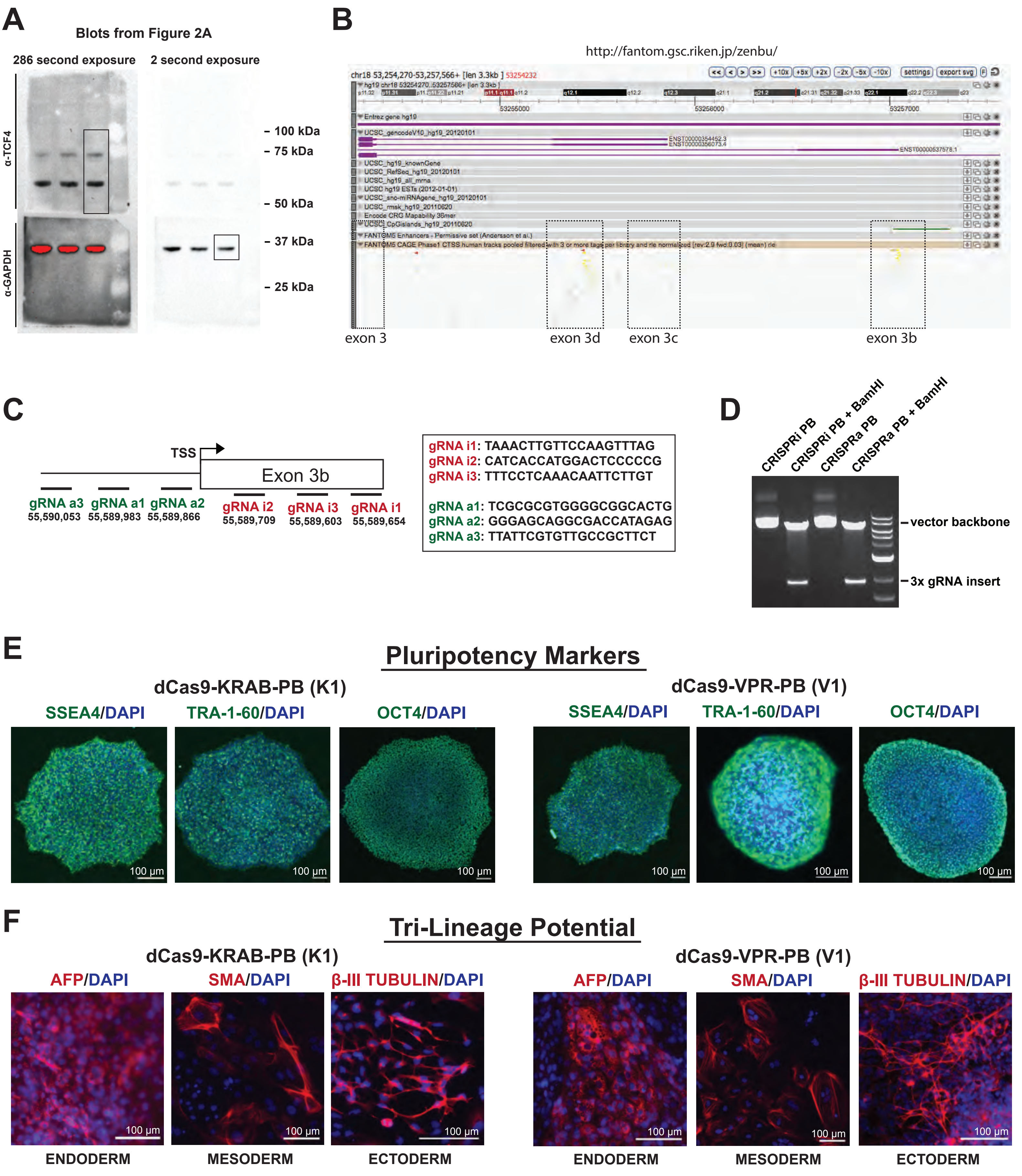
A
Blots from Figure 2A
286 second exposure
2 second exposure
α-TCF4
α-GAPDH
100 kDa
75 kDa
50 kDa
37 kDa
25 kDa
B
http://fantom.gsc.riken.jp/zenbu/
exon 3
exon 3d
exon 3c
exon 3b
C
TSS
Exon 3b
gRNA a3
55,590,053
gRNA a1
55,589,983
gRNA a2
55,589,866
gRNA i2
55,589,709
gRNA i3
55,589,603
gRNA i1
55,589,654
gRNA i1: TAAACTTGTTCCAAGTTTAG
gRNA i2: CATCACCATGGACTCCCCG
gRNA i3: TTTCCTCAAACAATTCTTGT
gRNA a1: TCGCGCGTGGGGCGGCACTG
gRNA a2: GGGAGCAGGCGACCATAGAG
gRNA a3: TTATTCGTGTTGCCGCTTCT
D
CRISPRi PB
CRISPRi PB + BamHI
CRISPRa PB
CRISPRa PB + BamHI
vector backbone
3x gRNA insert
E
Pluripotency Markers
dCas9-KRAB-PB (K1)
dCas9-VPR-PB (V1)
SSEA4/DAPI
TRA-1-60/DAPI
OCT4/DAPI
100 μm
F
Tri-Lineage Potential
dCas9-KRAB-PB (K1)
dCas9-VPR-PB (V1)
AFP/DAPI
SMA/DAPI
β-III TUBULIN/DAPI
100 μm
ENDODERM
MESODERM
ECTODERM

FIGURE S3

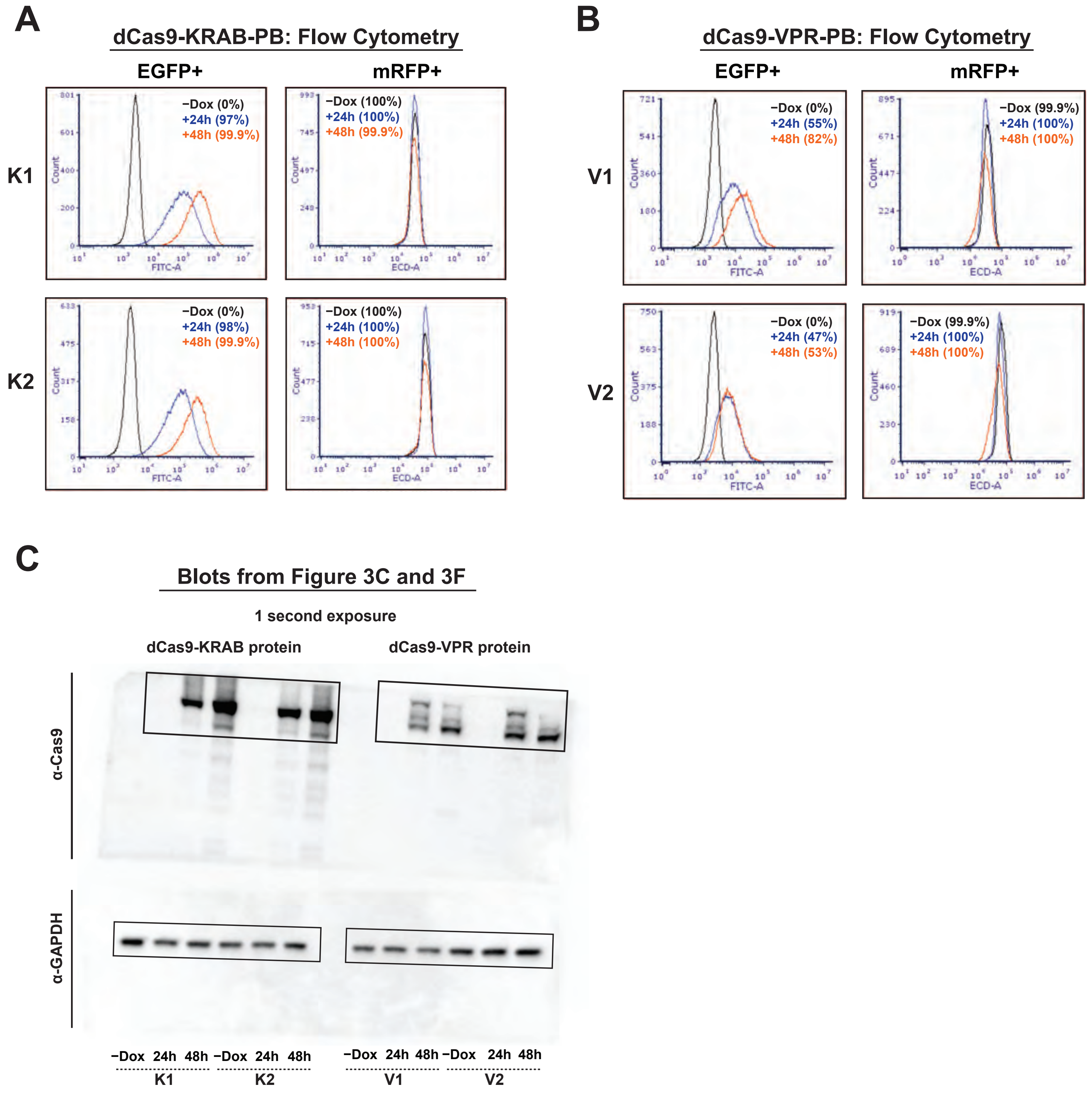
A
dCas9-KRAB-PB: Flow Cytometry
EGFP+
mRFP+
K1
−Dox (0%)
+24h (97%)
+48h (99.9%)
−Dox (100%)
+24h (100%)
+48h (99.9%)
K2
−Dox (0%)
+24h (98%)
+48h (99.9%)
−Dox (100%)
+24h (100%)
+48h (100%)
Count
FITC-A
ECD-A
B
dCas9-VPR-PB: Flow Cytometry
EGFP+
mRFP+
V1
−Dox (0%)
+24h (55%)
+48h (82%)
−Dox (99.9%)
+24h (100%)
+48h (100%)
V2
−Dox (0%)
+24h (47%)
+48h (53%)
−Dox (99.9%)
+24h (100%)
+48h (100%)
C
Blots from Figure 3C and 3F
1 second exposure
dCas9-KRAB protein
dCas9-VPR protein
α-Cas9
α-GAPDH
−Dox 24h 48h −Dox 24h 48h
K1 K2
−Dox 24h 48h −Dox 24h 48h
V1 V2

FIGURE S4

Blots from Figure 4B

Clone K1 - 3.4 second exposure

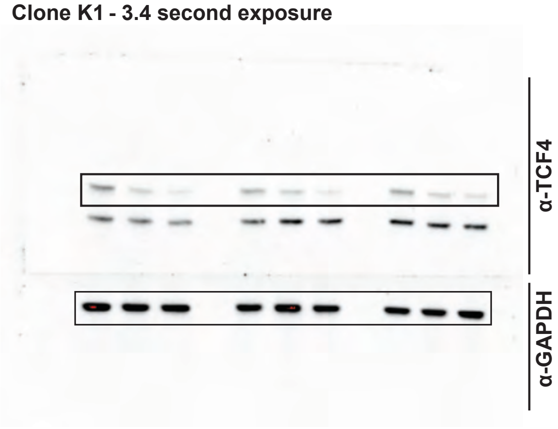

Clone K2 - 61.1 second exposure

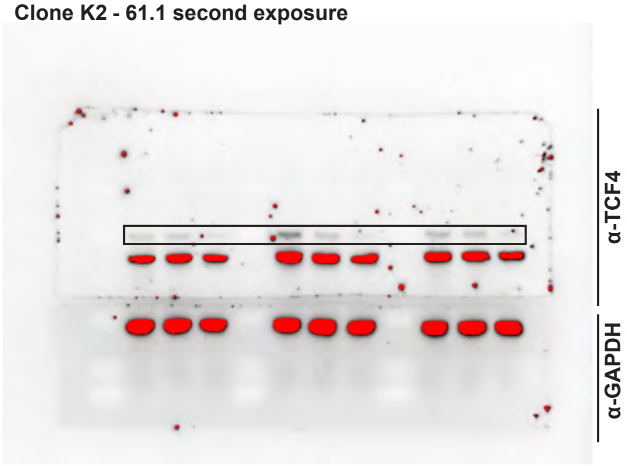

Clone K2- 7.0 second exposure

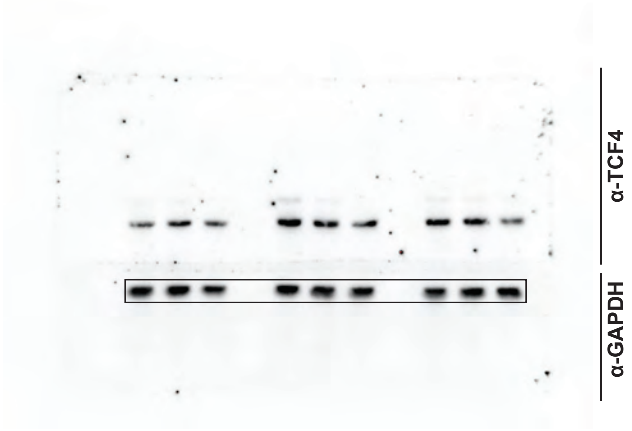

Blots from Figure 4D

Clone V1
2.2 second exposure

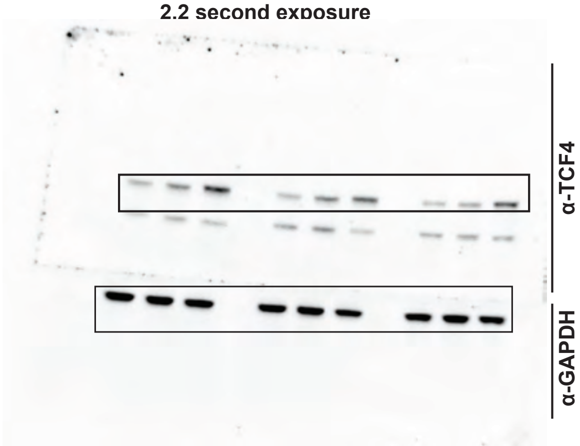

Clone V2
2.2 second exposure

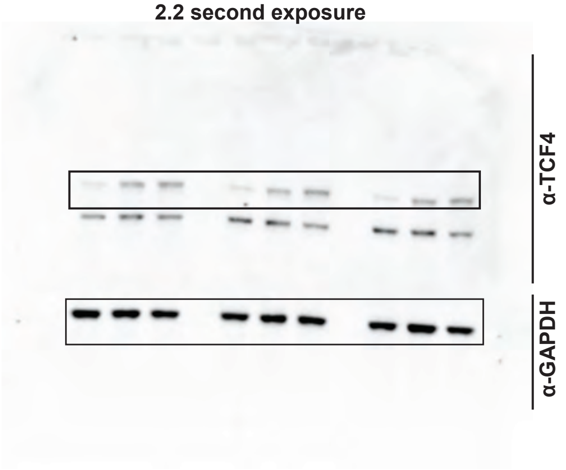

Blots from Figure 4F

TCF4 single guides

3.4 second exposure

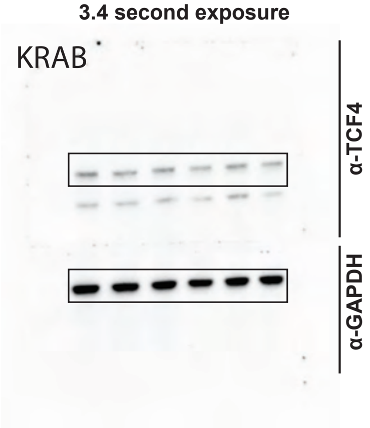

9.4 second exposure

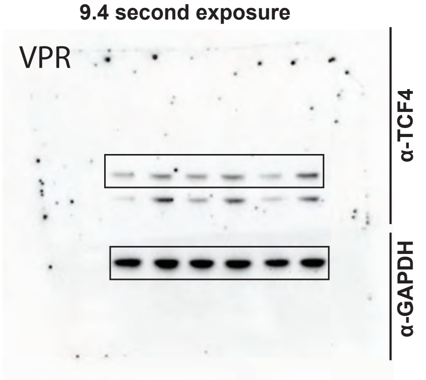

Blots from Figure 4G

POGZ single guides

2.2 second exposure

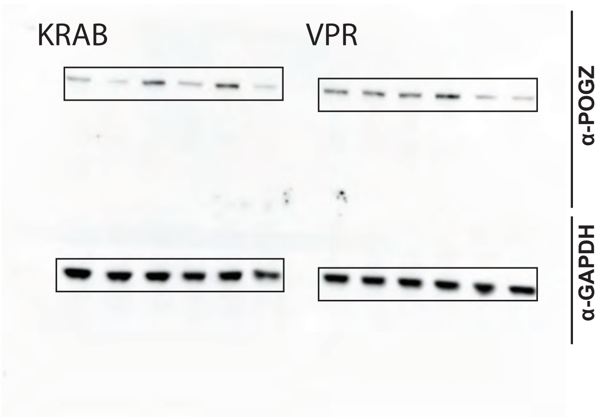

SUPPLEMENTARY FIGURE LEGENDS

Figure S1. A, B. Genotyping of AAVS1 integration in dCas9-KRAB and dCas9-VPR parental clones by junction PCR. **C.** *Left,* Flow cytometry plot of EGFP fluorescence in dCas9-KRAB cells after 48 hours of doxycycline treatment (+48h) followed by removal of doxycycline for 120 (-120h) and 168 hours (-168h) in comparison to no GFP control H1 cells (control). *Right,* representative image of EGFP expression in dCas9-KRAB cells after 48 hours doxycycline treatment. **D.** *Left,* Flow cytometry plot of EGFP fluorescence in dCas9-VPR cells after 48 hours of doxycycline treatment (+48h) followed by removal of doxycycline for 120 (-120h) and 168 hours (-168h) in comparison to no GFP control H1 cells (control). *Right,* representative image of EGFP expression in dCas9-VPR cells after 48 hours doxycycline treatment. E. Uncropped images from Figure 1D (*left*) and Figure 1E (*right*). Boxes denote cropped regions.

Figure S2. A. Uncropped gel blot images from Figure 2A. Boxes denote cropped regions. **B.** Results from FANTOM5 human promoterome depicting CAGE CTSS pooled signals for exons 3b, 3c, and 3d of *TCF4* gene. Exons are labeled according to nomenclature of Sepp et al.,[27]. **C.** Relative locations and sequences of *TCF4* gRNAs. Numbers correspond to genetic coordinates in hg38 human genome assembly. **D.** Confirmation of presence of 3x gRNA insert in multi-gRNA PB vectors by digestion with BamHI restriction enzyme. **E.** Representative immunostaining for pluripotency markers SSEA4, TRA-1-60, and OCT4 in dCas9-KRAB-PB and dCas9-VPR-PB cells. **F.** Representative immunostaining for AFP (endoderm), SMA (mesoderm) and β-III Tubulin (ectoderm) following embryoid body formation from dCas9-KRAB-PB and dCas9-VPR-PB cells. Cells are counterstained with DAPI.

Figure S3. A. Flow cytometry plots of EGFP and mRFP fluorescence in dCas9-KRAB-PB clones in absence of doxycycline (-Dox) and in presence of doxycycline for 24 (+24h) and 48 (+48h) hours. **B.** Flow cytometry plots of EGFP and mRFP fluorescence in dCas9-VPR-PB clones at indicated time-points. **C.** Uncropped gel blot images from Figures 3C (*left*) and 3F (*right*). Boxes denote cropped regions.

Figure S4. Uncropped gel blot images from Figures 4B (*top*), 4D (*middle*), 4F (*bottom* left) and 4G (*bottom right).* Boxes denote cropped regions.

SUPPLEMENTARY TABLE S1. Oligonucleotides and plasmids used in study.

OLIGONUCLEOTIDE NAME	SEQUENCE 5' to 3'	PURPOSE
GE_222_AAVS1_736 F	TCGACTTCCCCTCTTCCGAT	AAVS1 locus genotyping
GE668_AAVS1_PCR_D_Rev	ATGCAGGGGAACGGGGAT	AAVS1 locus genotyping
GE586_dCas9-VPR-EGFP_Seq_FOR_08	CATGATGGAGACCTTCTCAG	AAVS1 locus genotyping
GE332_TNTDNA308 Neo R1	TTCATCCTGCAGCTCGTTCA	AAVS1 locus genotyping
GE749_TCF4_classC_qPCR_A_F	TGGTACTCAGTCCTGCTCCA	qPCR of TCF4 Exon 3b
GE750_TCF4_classC_qPCR_A_R	GGAGGGAATTTTGTTGCAGT	qPCR of TCF4 Exon 3b
GE745_TCF4_classB_qPCR_A_F	AGTTCAGTTTTTGCCCGTTG	qPCR of TCF4 Exon 3c
GE746_TCF4_classB_qPCR_A_R	AGAAAGAAAGAAGTGAGGGGATG	qPCR of TCF4 Exon 3c
GE741_TCF4_classA_qPCR_A_F	AGGCGGCGTTCATGTCTA	qPCR of TCF4 Exon 3d
GE742_TCF4_classA_qPCR_A_R	CTGTGTGTCTGCGGATCTGT	qPCR of TCF4 Exon 3d
GE743_TCF4_classA_qPCR_B_F	AGAAGGGGCTCTCCGTG	qPCR of TCF4 Exon 3d
GE744_TCF4_classA_qPCR_B_R	CTGTGTGTCTGCGGATCTGTAGT	qPCR of TCF4 Exon 3d
GE897_TCF4_BB_TSS_qPCR_B_F	CAGAGCCTGCAAAAAGCAAAGG	qPCR of TCF4 Exon 4a
GE898_TCF4_BB_TSS_qPCR_B_R	GTAGCCCTAGGCAGGCA	qPCR of TCF4 Exon 4a
GE899_TCF4_SBon_TSS_qPCR_A_F	CCCCCAATATATCTGGTGATT	qPCR of TCF4 Exon 5b
GE900_TCF4_SBon_TSS_qPCR_A_R	ACAAGGAAGGCCCCTTAAAA	qPCR of TCF4 Exon 5b
GE903_TCF4_Drake_TSS_qPCR_A_F	GGGAGGCACCAGAAGATCTAA	qPCR of TCF4 Exon 7a
GE904_TCF4_Drake_TSS_qPCR_A_R	CACGCCACAACAGTTTATTCA	qPCR of TCF4 Exon 7a
GE907_TCF4_Sparrow_TSS_qPCR_A_F	GGCAATGTATGCAAGCAAGA	qPCR of TCF4 Exon 7b
GE908_TCF4_Sparrow_TSS_qPCR_A_R	TGGAAGTGTGGAGCAGTTTG	qPCR of TCF4 Exon 7b
GAPDH	QuantiTect, Qiagen cat# QT00079247)	qPCR of GAPDH
mRFP F	ATCTGAAGCTCTCCTTCCCT	ddPCR of mRFP
mRFP R	CTGGAGGGTGCTATCTTGTG	ddPCR of mRFP
mRFP probe	AACTTCGAGGACGGAGGCGT (Bio-Rad)	ddPCR of mRFP

PLASMID NAME	DESCRIPTION	REFERENCE
pPN454	U6 promoter-gRNA i1: TAAACTTGTTCCAAGTTTAG	This study
pPN434	U6 promoter-gRNA i2: CATCACCATGGACTCCCCCG	This study
pPN435	U6 promoter-gRNA i3: TTTCCTCAAACAATTCTTGT	This study
pPN446	U6 promoter-gRNA ai: TCGCGCGTGGGGCGGCACTG	This study
pPN431	U6 promoter-gRNA a2: GGGAGCAGGCGACCATAGAG	This study
pPN432	U6 promoter-gRNA a3: TTATTCGTGTTGCCGCTTCT	This study
pPN447	U6 promoter-gRNA POGZi: TCCTCGGGTCAGACTACCGG	This study
pPN455	U6 promoter-gRNA POGZa: TTGGGGAGACGAGCTTTCAA	This study
pPN441	3x gRNA i1,i2,i3 PB vector	This study
pPN440	3x gRNA a1,a2,a3 PB vector	This study
pPN459	1x gRNA i1 PB vector	This study
pPN458	1x gRNA a1 PB vector	This study
pPN461	1x gRNA POGZi PB vector	This study
pPN460	1x gRNA POGZa PB vector	This study

pT077	TRE3G-KRAB-dCas9-IRES-EGFP-CAG-TetON-NeoR-SA	This study
pT076	TRE3G-dCas9-VPR-T2A-EGFP-CAG-TetON-NeoR-SA	This study
AAVS1 Talen L	Addgene 59025	Gonzalez et al., 2014
AAVS1 Talen R	Addgene 59026	Gonzalez et al., 2014
PB-CA	Addgene 20960	Woltjen et al., 2009
pGEP150	PB transposase vector	System Biosciences #PB210PA-1
pGEP163	PB donor plasmid: mRFP-T2A-BlasticidinR	This study
pX330S	Vector for multi-gRNA cloning	Addgene #1000000055
pGEP179_pX330K	Entry vector for multi-gRNA cloning	This study
pGEP116	Ngn2 AAVS1 donor plasmid	Sellgren et al., 2019
pHR-TRE3G-KRAB-dCas9-IRES-GFP	Gift of Jesse Engrietz, Broad Institute	Fulco et al., 2016
SP-dCas9-VPR	Addgene 63798	Chavez et al., 2015
